# Effects of Early Fructooligosaccharides Intervention on Growth Performance and Jejunal Development in Suckling Piglets

**DOI:** 10.3390/ani16152332

**Published:** 2026-07-30

**Authors:** Zhuang Hao, Xuedong Ding, Yajun Gao, Zexu Li, Jing Wang

**Affiliations:** National Center for International Research on Animal Gut Nutrition, Jiangsu Key Laboratory of Gastrointestinal Nutrition and Animal Health, Laboratory of Gastrointestinal Microbiology, College of Animal Science and Technology, Nanjing Agricultural University, Nanjing 210095, China; 2022205038@stu.njau.edu.cn (Z.H.); 2021205031@stu.njau.edu.cn (X.D.); 2023105074@stu.njau.edu.cn (Y.G.); 2023105075@stu.njau.edu.cn (Z.L.)

**Keywords:** fructooligosaccharides, growth performance, jejunum, small intestine development, suckling piglets

## Abstract

Fructooligosaccharides (FOSs) are a class of functional prebiotics that effectively modulate the composition of the intestinal microbiota and regulate metabolic processes in animals. This study aimed to determine how early FOS supplementation affects jejunal development, which is vital for the rapid growth of suckling piglets. Sixty newborn piglets (Duroc × Landrace × Large White) from six litters were randomly assigned to either a control (CON) or an FOS group (*n* = 5 per litter per group). From days 2 to 21, piglets in the FOS group received 5 mL of an FOS solution orally (3 g/day/piglet), while those in the CON group received an equal volume of saline. Piglets were weighed on days 1, 7, 14, and 21, and samples were collected on days 7, 14, and 21. Early FOS intervention can enhance the growth performance of suckling piglets (*p* < 0.01), which is related to the development of the jejunum. Specifically, it improved intestinal morphology, stem cell function, and nutrient transport by increasing mRNA and protein expression of key genes in the Wnt/β-catenin signaling pathway. This may be related to increased abundance of jejunal *Lactobacillus* and the concentrations of metabolites such as lactate and short-chain fatty acids (SCFAs). Early FOS intervention drove jejunal development through Wnt/β-catenin activation, a process mediated by microbiota-metabolite shifts that augmented intestinal stem cell renewal.

## Simple Summary

The suckling period is a critical stage for intestinal development in young mammals, during which the gastrointestinal tract remains highly adaptive to environmental factors and is strongly influenced by nutrient intake and microbial communities. In this study, we used suckling piglets as an animal model and fructooligosaccharides (FOSs) as an early nutritional intervention to explore the effect and mechanism of FOSs on the development of the small intestine. Our findings suggest that there is a correlation between the supplementation of FOS and the activation of the Wnt/β-catenin signaling pathway, likely through the regulation of microbial composition and short-chain fatty acid levels in the jejunum of suckling piglets.

## 1. Introduction

The gastrointestinal tract performs essential functions in nutrient digestion, absorption, and metabolism, while also acting as a selective barrier that prevents the entry of harmful antigens, pathogens, and toxins [1,2]. The suckling period is a critical stage of intestinal development, during which the gastrointestinal tract adapts to environmental factors and is strongly influenced by nutrient intake and the microbial community [3,4]. Nutrition provides fuel for intestinal development and shapes the composition of intestinal microorganisms. Moreover, intestinal microorganisms and their secondary metabolites can influence the cell proliferation, structure and functional maturation of the intestine [4]. Nutritional intervention is a key strategy for promoting the rapid development of the piglets’ intestine.

Prebiotics are a relatively new form of nutritional intervention and have received increasing attention in recent years [5,6,7]. Fructooligosaccharides (FOSs) are a type of fructan oligosaccharide widely present in various plants. They are composed of 3–19 fructose units linked by β-(2,1) glycosidic bonds [8]. The beneficial effects of FOSs range from improving growth performance and gut health in suckling piglets to enhancing digestive function in fattening pigs by increasing short-chain fatty acid (SCFA) production and key enzyme activities [9,10,11,12]. Current research on FOSs has primarily focused on improving growth performance, intestinal tissue morphology and immunity of weaned piglets and fattening pigs [11,12]. However, research on the regulatory effects of FOSs on small intestine development, stem cell proliferation and differentiation, activity, and related mechanisms in suckling piglets remains limited.

This study aimed to test the hypothesis that FOSs enhance intestinal development in suckling piglets by determining how early supplementation influences growth performance, jejunal maturation, microbiota composition, metabolite profiles, and intestinal stem cell proliferation and differentiation.

## 2. Materials and Methods

### 2.1. Animal and Experimental Design

All experimental piglets and their sows were housed in the same shed, with each sow feeding 10 piglets. From 6 litters of piglets, within each litter, 10 piglets were randomly assigned to the CON group and the FOS group (FOS was purchased from Guangdong quantum high tech Biotechnology Co., Ltd., Jiangmen, Guangdong, China), with 6 replicates in each group and 5 piglets in each replicate, to ensure the balance of genetic background among the treatment groups. All experimental sows were multiparous, having given birth 2 to 4 times, with an average parity of 3.0 ± 0.5. All sows were fed the same feed. The number of piglets in each litter was the same. From days 2 to 21, piglets in the FOS group received 5 mL of an FOS solution orally (3 g of FOS dissolved in 5 mL of saline; 0.6 g/mL), while those in the CON group received 5 mL of saline. Piglets were weighed on days 1, 7, 14, and 21.

### 2.2. Sample Collection

During the experimental period, all piglets in each group were weighed and recorded on days 7, 14, and 21. At the same time, one piglet was randomly selected from the CON or FOS group in each litter, whose weight was closest to the average weight of that group, for euthanasia. Jejunal tissue, mucosa, and digesta samples were collected and immediately flash-frozen in liquid nitrogen.

### 2.3. Intestinal Morphometry

Mid-jejunal segments (2 cm) were collected, rinsed with saline, and fixed in 4% paraformaldehyde. After paraffin embedding, 5 μm sections were prepared and stained with hematoxylin and eosin (HE) for morphometric analysis. Villus height (VH) and crypt depth (CD) were measured in six randomly selected fields per section (×100 magnification) using ImageJ software 2024.

### 2.4. Jejunal Disaccharide Activities and Growth Factors

Jejunal mucosal supernatants were obtained from 10% homogenates centrifuged at 4000× *g* (4 °C, 15 min) and analyzed for total protein content using the bicinchoninic acid (BCA) method. Enzymatic activities (lactase, sucrase, and maltase) and concentrations of gut peptides glucagon-like peptide-1 (GLP-1), glucagon-like peptide-2 (GLP-2), insulin-like growth factor 1 (IGF-1), and epidermal growth factor (EGF) were measured using commercial kits (Nanjing Jiancheng, Nanjing, China). All assays were performed in triplicate.

### 2.5. RNA Extraction, cDNA Synthesis, and Real-Time RT-PCR for Gene Expression Analysis

Total RNA was extracted from 0.1 g of jejunal mucosa and reverse-transcribed into cDNA using a commercial kit (TaKaRa, Kusatsu, Shiga, Japan). Quantitative PCR (qPCR) was performed under standard cycling conditions. Gene expression levels were calculated according to the method of Livak et al. [13]. Primer sequences for the target genes are listed in Table S1. The relative mRNA expression of the target gene in each group was computed using the following formulas: ΔCt = Ct (target gene) − Ct (β-actin), and ΔΔCt = ΔCt (treated group) − ΔCt (CON group on day 7).

### 2.6. Immunohistochemistry

Paraffin-embedded intestinal sections were deparaffinized, rehydrated, and subjected to heat-induced antigen retrieval in citrate buffer (pH 6.0). Endogenous peroxidase activity was quenched using 3% H_2_O_2_, followed by blocking with normal goat serum. Sections were incubated overnight at 4 °C with primary antibodies (anti-Ki67, anti-PCNA, and anti-LGR5) and subsequently incubated with HRP-conjugated secondary antibodies for 1 h at room temperature. Signals were developed using diaminobenzidine (DAB), counterstained with hematoxylin, and visualized under a microscope at 400× magnification. The number of positive cells per crypt was quantified by assessing 30 well-preserved crypts.

### 2.7. 16S rRNA Gene Amplification and High-Throughput Sequencing

Jejunal digesta samples were initially homogenized in PBS and subjected to low-speed centrifugation. The supernatant was then centrifuged at 12,000× *g* for 10 min. After repeated washing, the pellet was resuspended in CTAB buffer and lysed mechanically. DNA was extracted using the phenol–chloroform–isoamyl alcohol method as described by Tremarol et al. [14]. The V3–V4 region of the bacterial 16S rRNA gene was amplified using primers 319F (ACTCCTACGGGAGGCAGCAG) and 806R (GGACTACHVGG GTWTCTAAT) [15]. PCR conditions were as follows: initial denaturation at 95 °C for 2 min; 25 cycles of 95 °C for 30 s, 55 °C for 30 s, and 72 °C for 30 s; followed by a final extension at 72 °C for 7 min. PCR products were analyzed using 2% agarose gel electrophoresis and normalized using an AxyPrep^TM^ Mag PCR Normalizer (Axygen, Union City, CA, USA). Subsequently, an Illumina PE250 library (San Diego, CA, USA) was constructed and quality-checked. The 16S rRNA sequencing data have been deposited in the NCBI Sequence Read Archive Database and are accessible under accession number PRJNA1406348. Sequencing was performed by Beijing Baimaike Bioinformation Technology Co., Ltd. (Beijing, China).

### 2.8. Determination of SCFAs and Lactate Concentrations in the Jejunum

Approximately 0.2 g of jejunal digesta was placed into a 2 mL sterile centrifuge tube, and the exact weight was recorded. Subsequently, 1.2 mL of deionized water was added, and the sample was vigorously mixed and centrifuged at 10,000 rpm at 4 °C for 10 min. One milliliter of the supernatant was deproteinized overnight with 200 μL of 25% metaphosphoric acid at −20 °C, followed by centrifugation at 13,000 rpm for 10 min at 4 °C. The resulting supernatant was filtered through a 0.22 μm membrane prior to analysis by gas chromatography [16].

### 2.9. Statistical Analysis

Data were analyzed using two-way ANOVA in SPSS 20.0, with diet, age, and their interaction included in the model. The experimental unit was the litter for growth performance parameters and the individual piglet for all other measurements. When significant interactions or main effects were detected (*p* < 0.05), means were compared using Duncan’s test. Litters were included as random effects in the statistical model, and age was included as a repeated measurement factor in the mixed model. The statistical model was set as follows: Fixed Effects = Diet + Age + Diet × Age Interaction. Immunohistochemical data were analyzed using Student’s *t*-test. Statistical significance was defined as *p* < 0.01 (highly significant), 0.01 ≤ *p* < 0.05 (significant), and 0.05 ≤ *p* < 0.10 (trend). All data are presented as mean ± SD.

## 3. Results

### 3.1. Effect of Early FOS Intervention on the Growth Performance and Intestinal Morphology of Suckling Piglets

**Figure 1:**
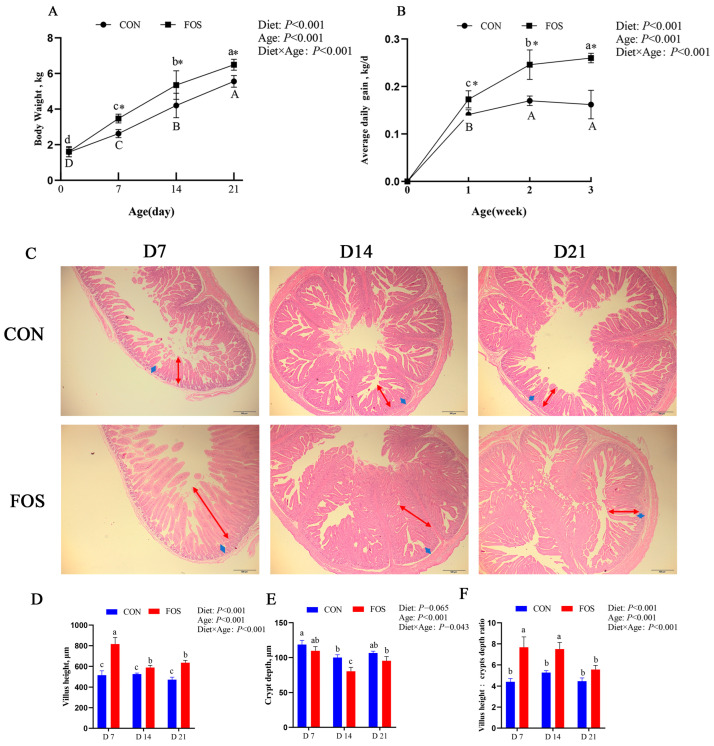
Effect of early FOS intervention on growth performance and intestinal morphology of suckling piglets. (**A**) Body weight of suckling piglets during the experimental period; (**B**) average daily gain of suckling piglets. Within each dietary group, different letters denote significant differences (*p* < 0.05) across ages (uppercase letters for the CON group and lowercase letters for the FOS group). * Indicates a significant difference (*p* < 0.05) between dietary groups at each age. Values are expressed as means ± SD. CON, control group; FOS, fructooligosaccharide group. (**C**) Histological morphology of the jejunum, the red arrow represents the height of villi, and the diamond represents the depth of crypt; (**D**) VH of the jejunum; (**E**) CD of the jejunum; (**F**) VH:CD ratio. Bars with different lowercase letters indicate significant differences.

Early FOS supplementation significantly modulated growth performance and intestinal development in suckling piglets (Figure 1). The interaction between diet and age significantly affected the growth performance and intestinal morphology of suckling piglets (*p* < 0.001). FOS significantly increased the body weight of suckling piglets at days 7, 14, and 21, as well as ADG during the first, second, and third weeks. In addition, FOS significantly increased the VH of the jejunum in suckling piglets on days 7, 14, and 21, significantly reduced CD on day 14, and increased the VH:CD ratio on days 7, 14 and 21. The indices of small intestinal organs are detailed in Table 1. The interaction between diet and age significantly affected small intestinal weight. The diet and age significantly affected the small intestinal length and weight (*p* < 0.001). FOS and age significantly increased the small intestinal length and weight of suckling piglets on days 7, 14, and 21.

### 3.2. Effect of Early FOS Intervention on Growth Factors in the Jejunum of Suckling Piglets

Supplementary Figure S1 shows the effects of early FOS on RNA expression and concentration of key intestinal growth factors (EGF, IGF1R) in the jejunum of suckling piglets. The mRNA expression and concentration of growth factors were significantly modulated: EGF, IGF1R and the mRNA expression of *IGF-1* and *IGF1R* by a diet × age interaction (*p* < 0.05); GLP-2, IGF-1 and the mRNA expression of *GLP2* and *EGF* by diet (*p* < 0.05). Early FOS intervention significantly increased the mRNA expression of *EGF* and *IGF1* in the jejunal mucosa of suckling piglets, and increased the mRNA expression of *GLP2* and *IGF1R* on days 14 and 21. Additionally, FOS significantly increased the mRNA expression of *GLP-1* and *IGF-1R* genes in the jejunum on days 14 and 21, and significantly increased the mRNA expression of *EGF* and *IGF1* genes in the jejunum on days 7, 14, and 21 of suckling piglets. FOS significantly increased the concentrations of EGF in the jejunum of suckling piglets on days 7, 14, and 21, GLP-2 on day 7, IGF-1R on days 14 and 21, and IGF-1 on day 21.

### 3.3. Effects of Early FOS Intervention on Jejunal Disaccharidase Activity and Nutrient Transporters in Suckling Piglets

The interplay between diet and age significantly affected the mRNA expression of the glucose transporter marker gene *SGLT1* and the amino acid marker gene *EAAC1* (*p* < 0.05) and showed a trend of influence on the sucrase enzyme activity and the mRNA expression of the protein hydrolysis marker gene *APA*; the significant interaction between diet and age indicates that the effect of FOS treatment is highly dependent on the age of the piglets. Early FOS intervention significantly increased the maltase activity, lactase activity and sucrase activity in the jejunum on day 7 of suckling piglets and significantly increased the intestinal sucrase activity in the jejunum on day 14 and the intestinal maltase activity in the jejunum on day 21 of suckling piglets. Early FOS intervention significantly increased the mRNA expression of *SGLT1*, *APA* and *PEPT1* in the jejunum on day 14, and significantly increased the mRNA expression of *EAAC1*, *APA* and *PEPT1* in the jejunum on day 21 of suckling piglets. as shown in Supplementary Figure S2.

### 3.4. Effect of Early FOS Intervention on the Differentiation and Proliferation Ability of the Jejunum in Suckling Piglets

**Figure 2:**
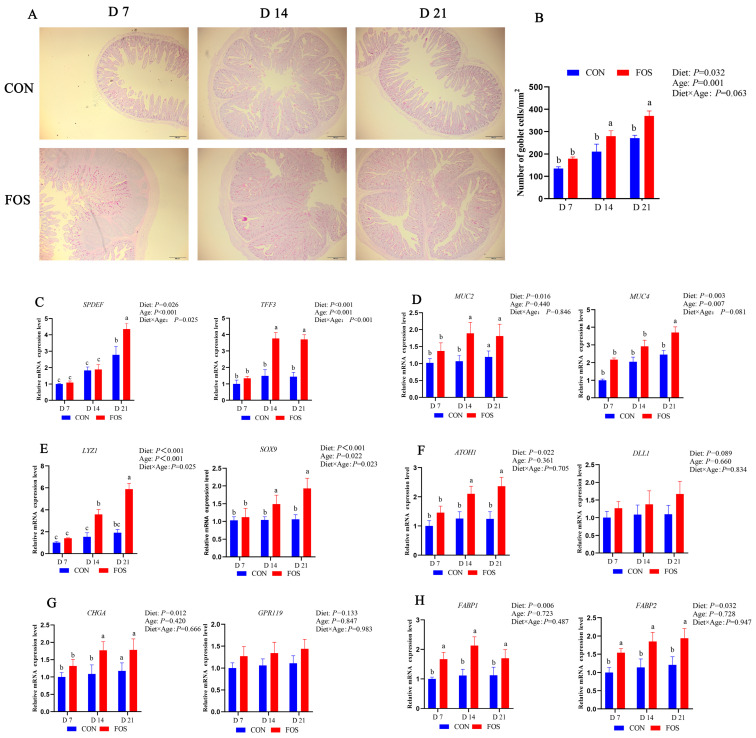
Effect of early FOS intervention on the differentiation capacity of the jejunum in suckling piglets. (**A**,**B**) Number of jejunal goblet cells; (**C**) expression of goblet cell marker genes; (**D**) expression of mucin marker genes; (**E**) expression of Paneth cell marker genes; (**F**) mRNA expression of intestinal secretory progenitor cell marker genes; (**G**) expression of enteroendocrine cell marker genes; (**H**) expression of maturation markers of absorptive epithelial cells. Data are presented as mean ± SD. Bars with different lowercase letters denote statistically significant differences.

The expression of marker genes for goblet cells and Paneth cells was significantly influenced by the interplay between diet and age (*p* < 0.05) and showed an influence trend with the number of goblet cells and the expression of mucin marker genes. A significant impact of the FOS diet was observed on the expression of marker genes related to secretory progenitor cells, secretory cells, and intestinal absorptive epithelial cells (*p* < 0.05). Early FOS intervention markedly raised the number of goblet cells in the jejunum on days 14 and 21. It also increased the mRNA expression of *SPDEF*, *MUC4*, and *LYZ1* on day 7, *TFF3*, *MUC2*, *SOX9*, *ATOH1*, and *CHGA* on day 14, and *FABP1* and *FABP2* in the jejunum of suckling piglets, as shown in Figure 2.

### 3.5. Effect of Early FOS Intervention on Proliferation Ability and Activity in the Jejunum of Suckling Piglets

**Figure 3:**
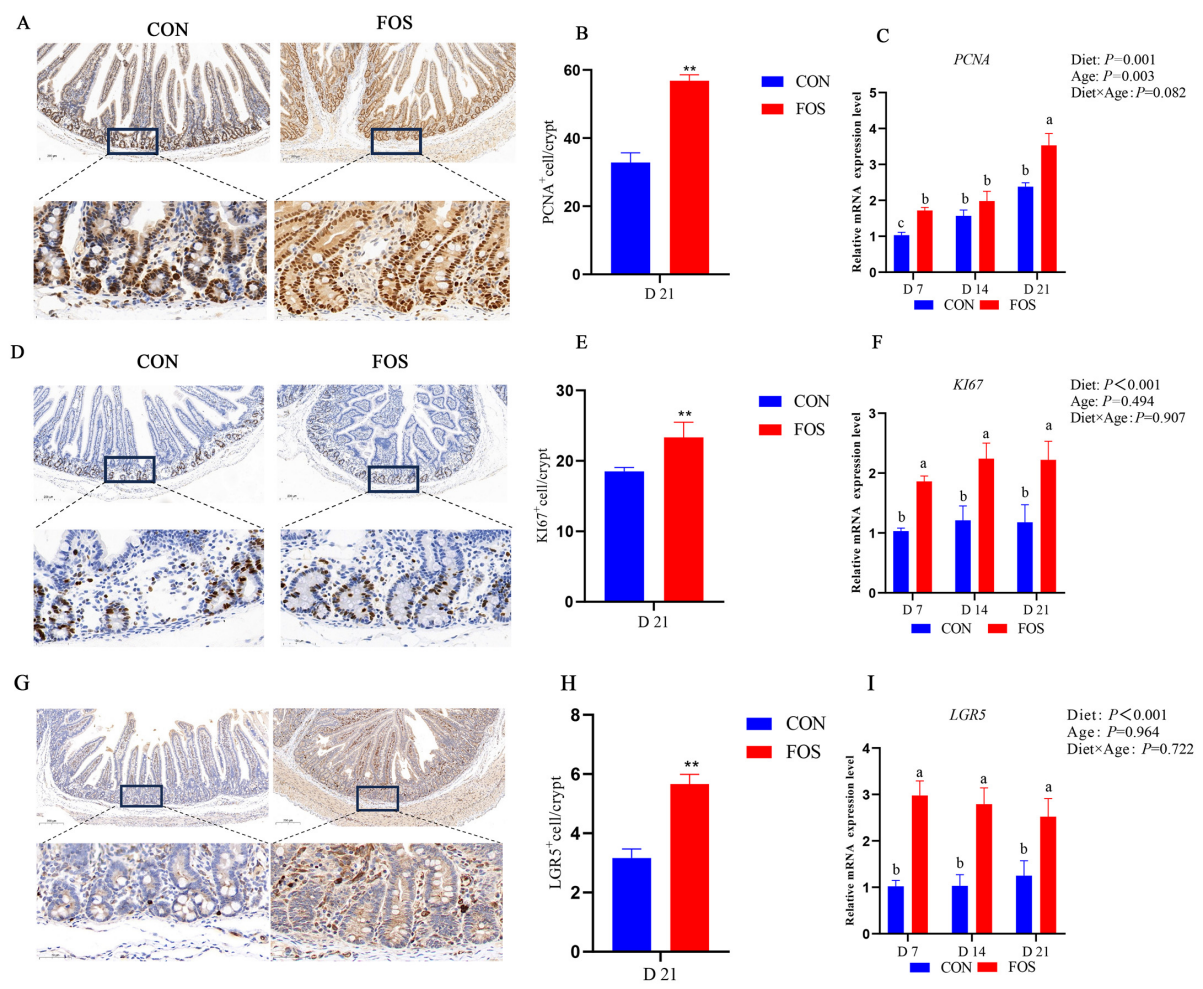
Effect of early FOS intervention on the proliferative capacity of the jejunum in suckling piglets. (**A**,**B**) Immunohistochemical analysis of PCNA; (**C**) PCNA mRNA expression by qPCR; (**D**,**E**) immunohistochemical analysis of Ki67; (**F**) Ki67 mRNA expression by qPCR; (**G**,**H**) immunohistochemical analysis of LGR5; (**I**) LGR5 mRNA expression by qPCR. Data are presented as mean ± SD. Different letters indicate significant differences among groups, while asterisks denote intergroup differences (** *p* < 0.01). Scale bars represent 50 μm (for ×200 images) and 200 μm (for ×400 images), respectively.

The relationship between diet and age showed a tendency to affect the mRNA expression of the proliferation marker gene *PCNA* in the jejunum of suckling piglets (*p* = 0.082). In contrast, the mRNA expression of the proliferation marker gene Ki67 in the jejunum was significantly affected by the FOS diet in suckling piglets (*p* < 0.001). FOS significantly increased the expression of *PCNA* and *Ki67* mRNA in the jejunum of suckling piglets. Immunohistochemical analysis revealed a greater number of PCNA- and Ki67-positive cells in the FOS group than those in the CON group. The FOS diet also markedly influenced the mRNA expression of the stem cell activity marker gene *LGR5* in the jejunum of suckling piglets. Early FOS intervention significantly increased the mRNA expression of *LGR5* in the jejunum of suckling piglets. According to immunohistochemical results, the FOS group exhibited considerably more LGR5-positive cells than the CON group, as shown in Figure 3.

### 3.6. Effect of Early FOS Intervention on the Expression of Key Genes and Proteins in the Wnt/β-Catenin Signaling Pathway in the Jejunum of Suckling Piglets

**Figure 4:**
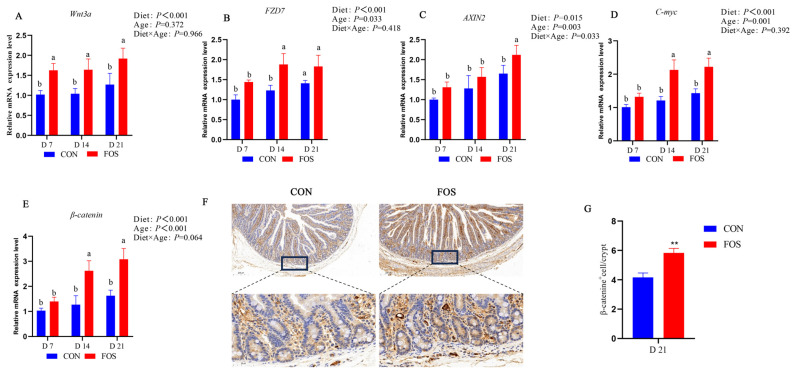
Effects of early FOS intervention on the mRNA expression and protein levels of key genes in the Wnt/β-catenin signaling pathway in the jejunum of suckling piglets. (**A**–**E**) qPCR analysis of key Wnt/β-catenin signaling pathway genes; (**F**,**G**) immunohistochemical analysis of β-catenin. Bars with different lowercase letters indicate statistically significant differences among groups. Asterisks denote intergroup differences (** *p* < 0.01). Scale bars represent 50 μm (for ×200 images) and 200 μm (for ×400 images), respectively.

The interplay between diet and age has a significant effect on the mRNA expression of the key gene *AXIN2* in the Wnt/β-catenin signaling pathway in the jejunum of suckling piglets and has a trend of affecting the mRNA expression of *β-catenin*. The FOS diet notably impacted the mRNA expression of key Wnt/β-catenin pathway genes including *Wnt3a*, *FZD7*, and *C-myc* in the jejunal tissue. Early FOS intervention significantly increased the mRNA expression of *AXIN2* in the jejunum on day 21, as well as the mRNA expression of *FZD7*, *C-myc*, and *β-catenin* in the jejunum on day 14. Immunohistochemical analysis further showed that the number of β-catenin-positive cells in the FOS group was significantly higher than that in the CON group, as shown in Figure 4.

### 3.7. Effect of Early FOS Intervention on Jejunal Microbial Diversity in Suckling Piglets

The effects of early FOS intervention on the microbial diversity of the jejunum are shown in Supplementary Figure S3. The interaction between diet and age significantly affected the Simpson index of the jejunal microbiota, and the FOS diet significantly influenced the Shannon index. The early FOS intervention significantly increased the Shannon index on days 14 and 21, and the Simpson index on days 7 and 21. The PCoA analysis results indicated that the jejunal microbial composition on days 7, 14, and 21 changed significantly in the FOS group compared with the CON group. A clear and statistically significant separation was observed between the CON and FOS groups (*p* < 0.05).

### 3.8. Effect of Early FOS Intervention on the Microbial Structure of the Jejunum in Suckling Piglets

**Figure 5:**
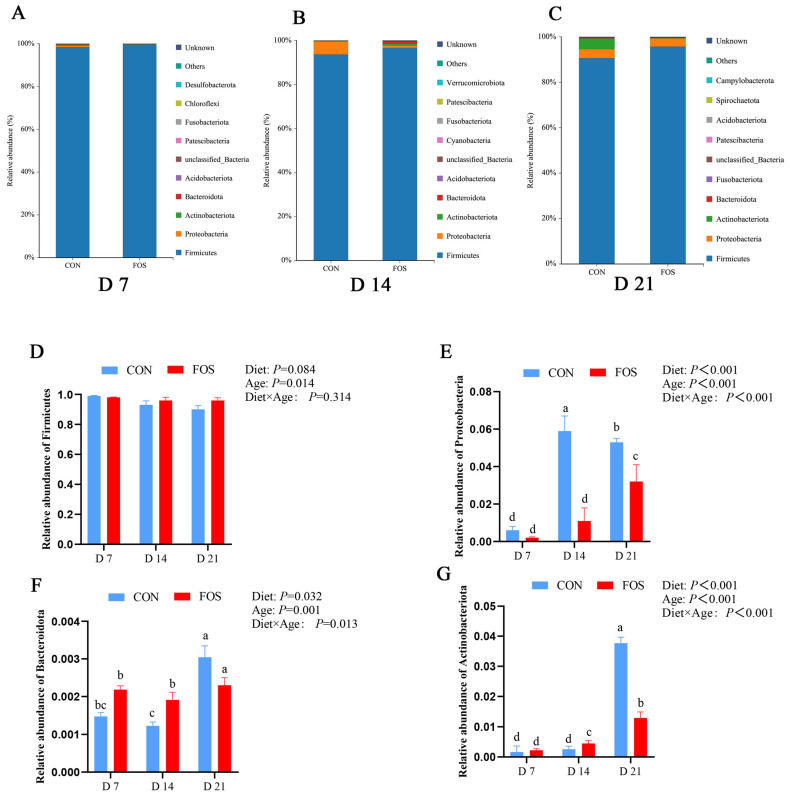
Effect of early FOS intervention on the phylum-level composition of the jejunal microbiota in suckling piglets. (**A**–**C**) Average relative abundance of jejunal microbiota in suckling piglets; (**D**–**G**) significantly altered phyla in jejunal digesta. Bars with different lowercase letters denote statistically significant differences.

Figure 5 illustrates the impact of early FOS intervention on microbial composition in the jejunum of suckling piglets at the phylum level. A significant interaction between diet and age was observed, influencing the relative abundances of Proteobacteria, Actinobacteria, and Bacteroidetes in the jejunum (*p* < 0.05). The FOS diet had a trend of influencing the relative abundance of Firmicutes. In comparison to the CON group, Firmicutes in the FOS group showed a gradually increasing trend with increasing age, while Proteobacteria showed a decreasing trend. FOS supplementation increased the relative abundance of Bacteroidetes but decreased that of Actinobacteria on days 7 and 14, and decreased the relative abundance of both Bacteroidetes and Actinobacteria on day 21 in the jejunum of suckling piglets.

**Figure 6:**
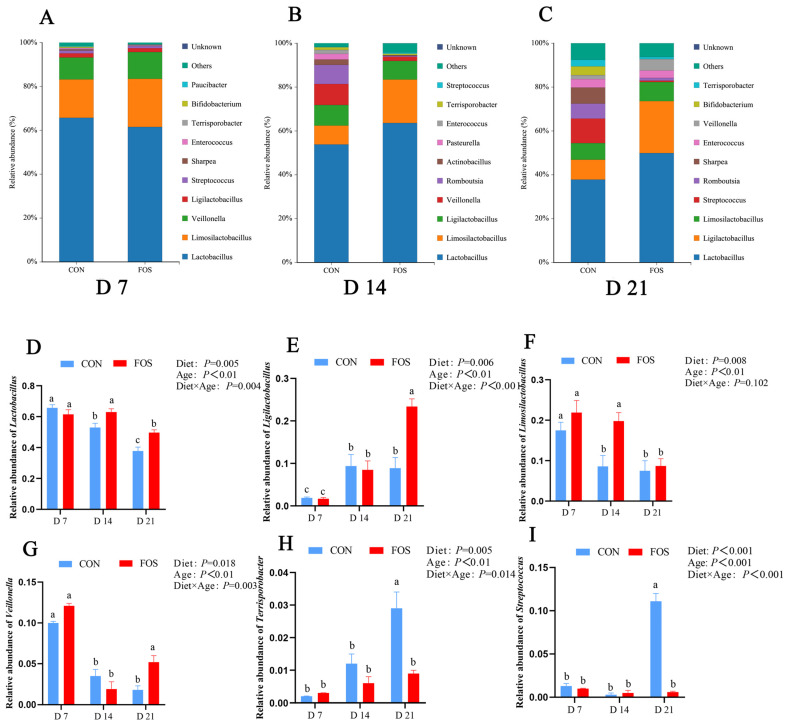
Effect of early FOS supplementation on the genus-level composition of the jejunal microbiota in suckling piglets. (**A**–**C**) Average relative abundance of jejunal microbiota at the genus level in suckling piglets; (**D**–**I**) genera showing significant changes in jejunal digesta. Bars with different lowercase letters denote statistically significant differences.

The interaction between diet and age significantly affected the relative abundance of *Lactobacillus*, *Ligilactobacillus*, *Veillonella*, *Streptococcus*, and *Terrisporobacter* in the jejunum of suckling piglets (*p* < 0.05). A significant effect of the FOS diet was observed on the relative abundance of jejunal *Limosilactobacillus* (*p* = 0.008). In comparison to the CON group, the relative abundance of *Lactobacillus* in the FOS group gradually increased with age, the relative abundance of *Limosilactobacillus* in the FOS group increased with age, and the relative abundance of *Ligilactobacillus* in the FOS group decreased first and then increased. FOS significantly increased the relative abundance of *Veillonella* on day 21, and significantly reduced the relative abundances of *Streptococcus* and *Terrisporobacter* on day 21 in the jejunum of suckling piglets, as shown in Figure 6.

### 3.9. Effects of Early FOS Intervention on the Concentrations of Lactate and SCFAs in the Jejunal Chyme of Suckling Piglets

The interaction between diet and age had a significant effect on the concentration of propionate in the jejunum of suckling piglets and had an influencing trend on the concentrations of lactate and acetate. A significant effect of the FOS diet was observed on the jejunal concentration of butyrate in suckling piglets. As shown in Table 2, early FOS intervention elevated lactate levels in the jejunal digesta on days 14 and 21. It also increased propionate concentration on day 21, as well as the concentrations of butyrate in the jejunal digesta on days 7, 14, and 21.

## 4. Discussion

This study investigated the effects of early FOS supplementation on growth performance, small intestinal development, and intestinal function in suckling piglets. The results demonstrated that early FOS intervention significantly influenced growth performance, small intestinal organ indices, jejunal morphology, jejunal digestive and absorptive functions, and epithelial proliferation and differentiation in the jejunum. Schokker et al. (2018) previously reported that supplementing suckling piglets with FOS for 12 days increased ADG, although the effect was not statistically significant [17]. In the present study, piglets received 3 g/day of FOS for 19 days, and early FOS supplementation significantly increased body weight and ADG, consistent with the trend observed by Schokker et al. (2018) [17]. The small intestine is the primary site of nutrient digestion and absorption in piglets. Its structural integrity is a key determinant of absorptive capacity and thus plays a fundamental role in overall growth and development [18]. Early FOS intervention significantly increased VH in the jejunum, reduced CD at day 14, and improved the VH:CD ratio, indicating enhanced nutrient absorption efficiency in suckling piglets. Similarly, Xu et al. (2005) demonstrated that dietary supplementation with 4% FOS during the weaning period significantly increased small intestinal VH in piglets [11]. These findings are consistent with the present results, confirming that FOS improves jejunal morphology in suckling piglets.

It is well established that growth factors exert significant regulatory effects on multiple physiological processes in suckling piglets, including nutrient digestion and absorption, gastrointestinal tract development, and growth performance [18,19,20,21]. In this study, early FOS supplementation significantly increased the mRNA expression of key growth factors in the jejunal mucosa. The corresponding protein levels showed patterns consistent with gene expression, suggesting that transcriptional regulation contributes to these effects. Efficient nutrient digestion and absorption depend largely on intestinal digestive enzymes and nutrient transporters in the jejunum [22,23]. Early FOS intervention significantly increased jejunal maltase activity, enhanced lactase activity at day 7, and increased sucrase activity at days 7 and 14. In addition, FOS increased the mRNA expression of genes related to glucose transport, proteolysis, and amino acid absorption in the jejunum of suckling piglets.

Intestinal growth factors are primarily secreted by Paneth cells and endocrine cells located within intestinal crypts, while disaccharidases and nutrient transporters are produced by mature absorptive epithelial cells [24,25]. Intestinal stem cells residing in the crypts give rise to these cell types through tightly regulated processes of proliferation and differentiation, thereby maintaining epithelial renewal [26]. These cell populations are essential for intestinal development [26]. In the present study, early FOS supplementation markedly increased the mRNA expression of markers associated with Paneth cells, intestinal secretory progenitor cells, secretory cells, mature absorptive epithelial cells, goblet cells, and mucin-related genes, and also increased the number of goblet cells in the jejunum of suckling piglets. These results indicate that FOS substantially enhanced the differentiation potential of jejunal stem cells in suckling piglets. These results indicate that FOS enhanced the differentiation potential of jejunal stem cells in suckling piglets. At the same time, these could be the reasons for the up-regulated mRNA expression of intestinal growth factors, disaccharidase activity, and nutrient transporter in the FOS group.

We further investigated jejunal proliferative capacity and the expression of stem cell activity markers. *PCNA* and *Ki67* are widely recognized markers of cellular proliferation [27,28]. Both qPCR and immunohistochemical analyses revealed a marked increase in the mRNA and protein expression levels of PCNA and Ki67 following FOS supplementation. Intestinal stem cells possess the ability to self-renew and generate specialized progenitor cells [29], a process that underlies epithelial proliferation and differentiation and supports rapid intestinal epithelial turnover [23,30,31]. These findings demonstrate that FOS supplementation significantly enhanced the activity, proliferative capacity, and differentiation potential of the jejunum in suckling piglets.

The Wnt/β-catenin signaling cascade is pivotal for the proliferation and differentiation of intestinal stem cells, driving intestinal growth by activating their proliferation and differentiation [32,33,34]. The initiation of the Wnt/β-catenin signaling cascade depends on the interaction between the primary ligand Wnt3a and the FZD7 receptor. This binding event enhances β-catenin expression and triggers the activation of downstream target genes, including *C-myc*, *AXIN2*, and *LGR5*, and the proliferation and differentiation processes of intestinal stem cells are stimulated [35,36,37,38,39]. Early supplementation with FOS significantly increased the mRNA expression of key genes in the jejunum Wnt/β-catenin signaling pathway, including *Wnt3a*, *FZD7*, *AXIN2*, *C-myc*, and *β-catenin*. Immunohistochemical analysis further revealed increased numbers of β-catenin- and LGR5-positive cells in the FOS group. These results indicate that early FOS administration increased the mRNA and protein expression of key genes in the Wnt/β-catenin signaling pathway, thereby enhancing jejunal epithelial proliferation and differentiation.

The intestinal microbiota and its secondary metabolites are key factors influencing the proliferation and differentiation of intestinal stem cells and the activation of the Wnt/β-catenin signaling pathway [36,40]. The fermentation of FOS by intestinal microbiota generates SCFAs and lactate, and modulates the composition of intestinal microbial communities [41,42]. In the present study, we investigated the impact of early FOS supplementation on jejunal microbiota across multiple taxonomic levels. Firmicutes emerged as the predominant bacterial group at the phylum level. In comparison to the CON group, Firmicutes in the FOS group showed a gradual increase with age. Proteobacteria in the FOS group showed a decrease with increased age, which mainly includes potential conditioned pathogens [43]. At the genus level, *Lactobacillus* and *Limosilactobacillus* showed age-dependent increases in the FOS group compared with the CON group. Notably, FOS supplementation significantly increased the relative abundance of *Ligilactobacillus* on day 21, while reducing the abundance of *Streptococcus* in the jejunum of suckling piglets. *Lactobacillus* represents the most extensive genus among lactate-producing bacteria and serves as the predominant microorganism in the jejunum of suckling piglets. It has the capacity to enhance the mRNA expression of Ki67 and PCNA in intestinal organoids [44,45,46]. Lactate-producing bacteria such as *Lactobacillus plantarum* and *Lactobacillus reuteri* activated the Wnt/β-catenin signaling pathway, up-regulated the mRNA expression of *Ki67*, *LGR5*, and *PCNA*, and improved the growth performance of piglets [47,48,49,50,51,52,53]. Similarly, acetate, propionate, butyrate, and valerate have been shown to enhance the expression of Ki67, LGR5, and PCNA at both the mRNA and protein levels in intestinal organoids [34,54,55,56]. In the present study, early FOS supplementation markedly elevated the levels of lactate, propionate, butyrate, and valerate in the jejunum. It also substantially activated the Wnt/β-catenin signaling pathway and concurrently up-regulated genes associated with intestinal proliferation, differentiation, and functional activity. Collectively, these findings indicate that FOS modulates the jejunal microecology of suckling piglets, thereby activating the Wnt/β-catenin signaling pathway and promoting intestinal stem cell proliferation, differentiation, and overall jejunal development.

## 5. Conclusions

Early FOS intervention significantly enhanced growth performance and improved jejunal morphology and digestive function. Furthermore, FOS can increase the mRNA and protein expression of key genes in the Wnt/β-catenin signaling pathway, as well as marker genes for stem cell proliferation and differentiation, which is closely related to changes in the jejunal microbiota and its metabolites (lactate and SCFAs). These effects collectively promoted the development of the jejunum and improved overall growth performance.

## Figures and Tables

**Table 1:** Effects of early FOS intervention on small intestine organ indices in suckling piglets.

Items	D7		D14		D21		SEM	*p*-Value		
	CON	FOS	CON	FOS	CON	FOS		Diet	Age	Diet × Age
SI length, cm	649.25 ^c^	788.67 ^b^	800.67 ^b^	947.25 ^a^	895.83 ^b^	968.83 ^a^	8.47	<0.001	<0.001	0.193
SI weight, g	125.33 ^c^	163.00 ^b^	176.83 ^b^	251.50 ^a^	220.83 ^b^	257.50 ^a^	4.25	<0.001	<0.001	0.048
SI weight/BW, g/kg	47.83	46.79	42.67	49.49	39.77	38.90	1.18	0.493	0.063	0.314

Note: Distinct letters on the shoulder labels denote significant differences, whereas identical or absent letters on the shoulder labels denote no significant differences.

**Table 2:** Effects of early FOS intervention on the concentration of lactate and short-chain fatty acids in jejunal digesta of suckling piglets.

Items	D7		D14		D21		SEM	*p*-Value		
	CON	FOS	CON	FOS	CON	FOS		Diet	Age	Diet × Age
Lactate µmol/g digesta	7.21 ^b^	8.36 ^b^	7.29 ^b^	10.10 ^a^	8.45 ^b^	11.40 ^a^	0.18	<0.001	<0.001	0.091
Acetate µmol/g digesta	1.99	2.00	2.76	3.09	4.20	4.52	0.32	0.997	0.032	0.761
Propionate µmol/g digesta	0.35 ^b^	0.24 ^b^	0.54 ^b^	0.79 ^b^	0.58 ^b^	1.21 ^a^	0.08	0.033	0.002	0.072
Butyrate µmol/g digesta	0.48 ^b^	0.84 ^a^	0.42 ^b^	0.82 ^a^	0.47 ^b^	0.91 ^a^	0.07	0.010	0.963	0.936
Valerate µmol/g digesta	0.15 ^c^	0.44 ^a^	0.26 ^c^	0.47 ^a^	0.32 ^c^	0.68 ^a^	0.01	<0.001	<0.001	0.036

Note: Different letters on the shoulder labels indicate significant differences, while the same or no letters on the shoulder labels indicate no significant differences.

## Data Availability

The original contributions presented in this study are included in the article/Supplementary Materials. Further inquiries can be directed to the corresponding author.

## Supplementary Materials

The following supporting information can be downloaded at: https://www.mdpi.com/article/10.3390/ani16152332/s1, Table S1: Gene primer sequences; Figure S1: Effects of early FOS intervention on growth factor mRNA expression and concentration in jejunal mucosa of suckling piglets. (A–D) Jejunal mucosal growth factor mRNA expression; (E–H) Concentration of growth factors in the jejunal mucosa; Values are expressed as means ± SD. Bars assigned with different lower-case letters indicate significant differences; Figure S2: Effects of early FOS intervention on jejunal disaccharase activity and mRNA expression of nutrient transporter related genes in suckling piglets. (A) Maltase activity; (B) Lactase activity; (C) Sucrase activity (D,E) Glucose transport; (F,G) Proteolysis; (H,I) Amino acid hydrolysis; Values are expressed as means ± SD. Bars assigned with different lower-case letters indicate significant differences; Figure S3: Effect of early FOS intervention on jejunal microbial diversity in suckling piglets; (A–D) Microbial diversity of the jejunum; (E–G) PcoA analysis of jejunal microorganisms. Bars assigned with different lower-case letters indicate significant differences.

## Abbreviations

The following abbreviations are used in this manuscript:

ADG	average daily gain
AXIN2	Axis Inhibition Protein 2
APA	Aminopeptidase A
APN	aminopeptidase N
BCA	Bicinchoninic acid
CHGA	Chromogranin A
CD	Crypt Depth
FOS	Fructooligosaccharide
KI67	Nuclear antigen KI67
MUC2	Mucin 2
MUC4	Mucin 4
Wnt3a	Wingless-type 3a
