# Supplementary material for: Effects of Early Fructooligosaccharides Intervention on Growth Performance and Jejunal Development in Suckling Piglets
Source: Animals (Basel). 2026 Jul 30;16(15):2332. doi: 10.3390/ani16152332 (PMC13463474; doi:10.3390/ani16152332)
Supplement: Supplementary file 1 [file animals-16-02332-s001.zip › animals-4436032-supplementary.pdf]

Effects of Early Fructooligosaccharides Intervention on Growth Performance and Jejunal Development in Suckling Piglets

Zhuang Hao, Xuedong Ding, Yajun Gao, Zexu Li and Jing Wang *

Supplementary Table S1. Gene primer sequences

Gene	Primer pairs sequence	Product length	Accession number
β-actin	F: CTGGAACGGTGAAGGTGA<br>R: TTTGGAAAGGCAGGGACT	109	XM_003124280
APA	F: GTCTCTACCACCTGACGAT<br>R: CTCTGTAAGTGATGAGTCC	555	NM_214017.1
APN	F: ACATCACTCTCATCCACCCT<br>R: GCAATCACAGTGACAACTCG	460	XM_005653524.3
EAAC1	F: ATAGAAGTTGAAGACTGGGAAAT<br>R: GTGTTGCTGAACTGGAGGAG	488	NM_001164649.1
SGLT1	F: CCACTTTCCCTATAAAACCTCAC<br>R: CTCCATCAAACTTCCATCCTCAG	151	NM_001164021.1
GLUT2	F: CCTGCTTGGTCTATCTGCTGTG<br>R: TTGATGCTTCTTCCCTTTCTTT	194	NM_001097417.1
PEPT1	F: CATCGCCATACCCTTCTG<br>R: TTCCCATCCATCGTGACATT	109	NM_214347.1
IGF-1	F: CCTGCGCAATGGAATAAAGTC<br>R: GCAAAGTCTGGAAATGAATTGGT	120	XM_021091140.1
IGF-1R	F: GGGATGACGAGAGACATCTATGAG<br>R: GAAGGACCAGACTCAGAGTGC	131	XM_021082920.1
EFG	F: ATCTCAGGAATGGGAGTCAACC<br>R: TCACTGGAGGATGGAATACAGC	166	XM_021100462.1
GLP-2	F: ACTCACAGGGCACGTTTACCA<br>R: AGGTCCCTTCAGCATGTCTCT	150	XM_005671883.3

LYZ1	F: AACTGCTTTGGGTGTCTTGC<br>R: GGTCTATGATCGGTGCGAGT	212	NM_013590
SOX9	F: CGGTTCGAGCAAGAATAAGC<br>R: GTAATCCGGGTGGTCCTTCT	150	NM_001244512.1
ATOH	F: TGAGAAGGAGCGGCGGTGTC<br>R: GCCTTTCAGCTTGCACAGTTGTTC	223	XM_021091011.1
DLL1	F: TGTGAGACAGAGGTGGACGAGTG<br>R: GTCCGCACACGCCATAGCAC	154	XM_021086798.1
CHGA	F: AGAAGAACGATGAAGACGCAGACG<br>R: TGCTGGGAAGGCTGGCTAAGG	120	XM_021091314.1
GPR119	F: CCACGCACAACCAGCAGATCC<br>R: GCCACAGTTCGGACAGCCTTG	186	XM_005664539.4
FABP1	F: GCCCGACGAACTCATCCAGAAG<br>R: GGTCTCCATCTCACACTCCTCTCC	142	XM_021075951.1
FABP2	F: GCCTGGAAGATAGACCGCAATG<br>R: TCCAATTCCCAGTGAGTTCAGTTC	153	XM_021091372.1
SPDEF	F: CCGCTTCATCAGGTGGCTCAAC<br>R: AGTTCATGGCAGGACGGTTCTTG	172	XM_021090146.1
TFF3	F: TGGCTGCTAGTGGTGCTCCTG<br>R: GCACACTGGTTCGCCGACAG	148	XM_021091621.1
MUC2	F: CTGCTCCGGGTCCTGTGGGA<br>R: CCCGCTGGCTGGTGCGATAC	132	XM_021082584.1
MUC4	F: GATGCCCTGGCCACAGAA<br>R: TGATTCAAGGTAGCATTCATTTGC	189	NM_001206344.2
PCNA	F: TACGCTAAGGGCAGAAGATAATG<br>R: CTGAGATCTCGGCATATACGTG	162	XM_021068219.1
KI67	F: GCAGGCTTAATGGCGGCTCTC<br>R: GTTTCTCTGGTTGCTTGGTTGCG	158	XM_021092028.1
OLFM4	F: GCTCACAACCACATCAGGAGGAAG	121	XM_021089949.1

	R: TCTGCCAACTGCCATTAGCTTCTG		
BMI1	F: ACTCCAGTGCAGTCTCCTCATCC<br>R: AAGTTGCTGACGACCCATTTACGG	187	XM_021085180.1
LGR5	F: CCTTGGCCCTGAACAAAATA<br>R: ATTTCTTTCCCAGGGAGTGG	152	XM_021085541.1

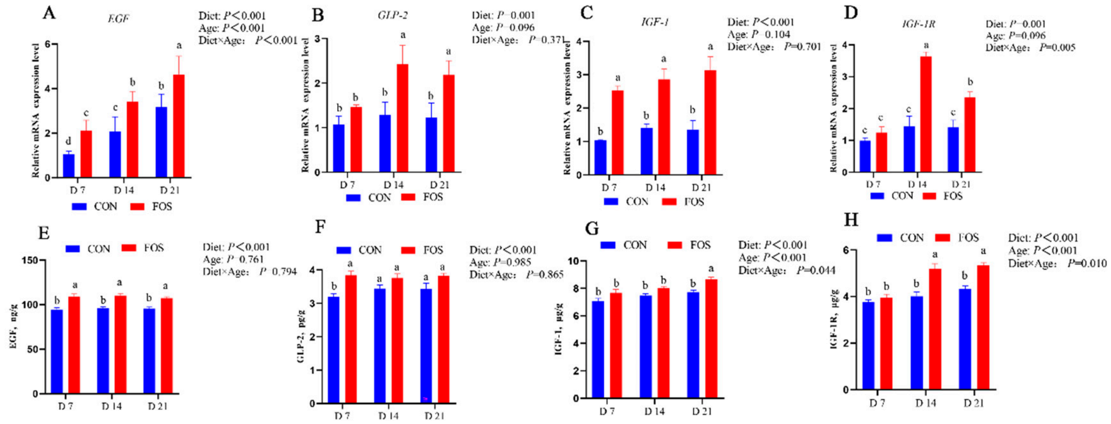

Supplementary Figure S1. Effects of early FOS intervention on growth factor mRNA expression and concentration in jejunal mucosa of suckling piglets. (A-D) Jejunal mucosal growth factor mRNA expression; (E-H) Concentration of growth factors in the jejunal mucosa; Values are expressed as means ± SD. Bars assigned with different lower-case letters indicate significant differences.

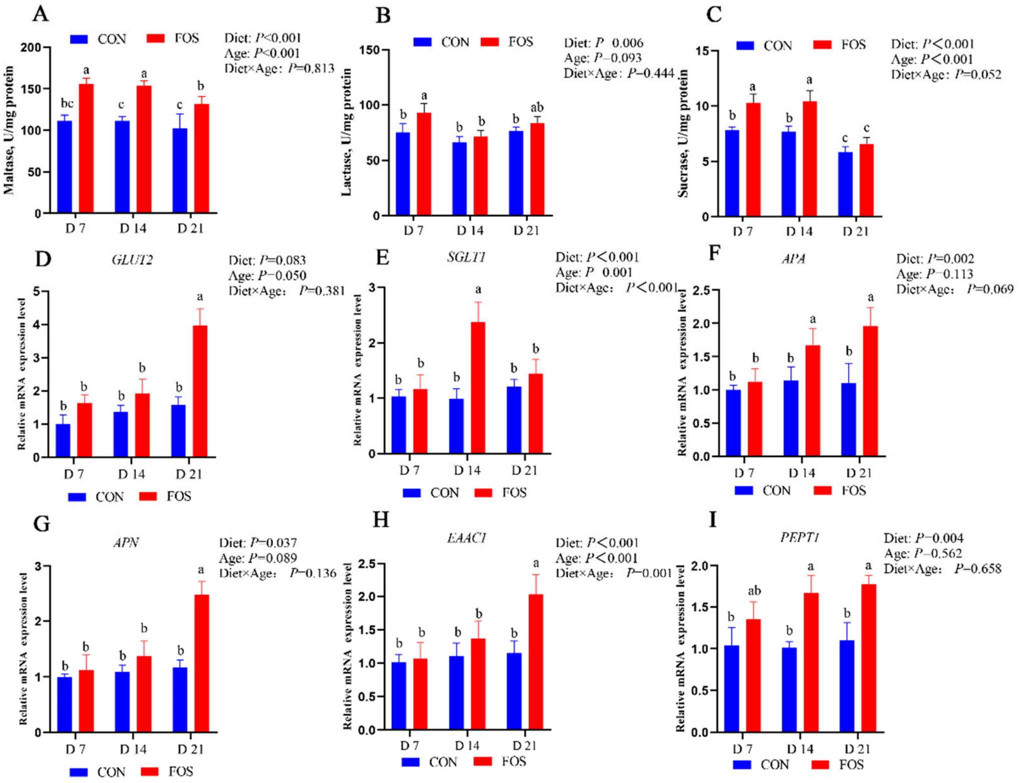

Supplementary Figure S2. Effects of early FOS intervention on jejunal disaccharase activity and mRNA expression of nutrient transporter related genes in suckling piglets. (A) Maltase activity; (B) Lactase activity; (C) Sucrase activity (D-E) Glucose transport; (F-G) Proteolysis; (H-I) Amino acid hydrolysis; Values are expressed as means ± SD. Bars assigned with different lower-case letters indicate significant differences.

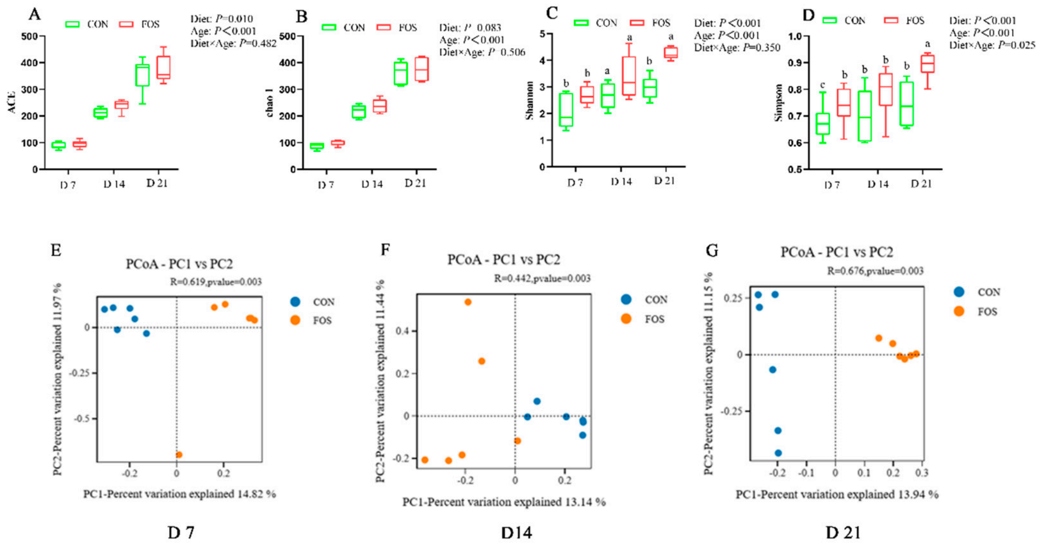

Supplementary Figure S3. Effect of early FOS intervention on jejunal microbial diversity in suckling piglets;(A-D) Microbial diversity of the jejunum；(E-G) PcoA analysis of jejunal microorganisms. Bars assigned with different lower-case letters indicate significant differences.
